# Mental health professionals’ experiences transitioning patients with anorexia nervosa from child/adolescent to adult mental health services: a qualitative study

**DOI:** 10.1186/s12913-020-05740-2

**Published:** 2020-09-21

**Authors:** Veronica Lockertsen, Liv Nilsen, Lill Ann Wellhaven Holm, Øyvind Rø, Linn May Burger, Jan Ivar Røssberg

**Affiliations:** 1 Division of Mental Health and Addiction, Oslo University Hospital, P.O. box 4959, Oslo, Nydalen Norway; 2 Institute of Clinical Medicine, Faculty of Medicine, University of Oslo, 0318 Oslo, Norway; 3 Melkeveien 33, 0779 Oslo, Norway; 4 Regional Department for Eating Disorders, Division of Mental Health and Addiction, Oslo University Hospital, Ullevål HF, Postboks 4950, 0424 Oslo, Nydalen Norway; 5 Formoenga 25, 3340 Amot, Norway

**Keywords:** Anorexia nervosa, Mental health transition, Adolescent, Professionals’ perspectives, Qualitative research

## Abstract

**Background:**

The transition period between child and adolescent mental health services (CAMHS) and adult mental health services (AMHS) has been identified as an especially critical time for patients with anorexia nervosa. In the present study, to better facilitate patients’ recovery process, we explored the experiences of professionals concerning the transition from CAMHS to AMHS.

**Method:**

A qualitative explorative study was carried out based on recorded interviews from one multi-step focus group and two individual interviews with eight experienced health care professionals. Together they had experience with treating patients with AN and the transition from CAMHS to AMHS, both from specialized eating disorder units, specialized mental health care units, and from a school nurse context. Service users with parents` perspectives and patients’ perspectives were involved in all steps of the research process.

**Results:**

Barriers experienced during the transition process were classified into four categories: (1) different treatment cultures that describe differences in how parents are included in CAMHS and AMHS; (2) mistrust between CAMHS and AMHS that can create a lack of collaboration and predictability for the patients’ transition; (3) Clinicians` factors such as lack of professional self-confidence can influence continuity of care for patients; and (4) lack of trust between services and not enough focus on building a new alliance in AMHS negatively influences the transition.

**Conclusions:**

The present study revealed four important categories that professionals needs to consider when participating in the transition for patients with AN from CAMHS to AMHS. Awareness of these challenges might improve the transition process for patients with AN.

## Background

Adolescents with mental health disorders who receive care in child and adolescent mental health services (CAMHS) often need further treatment in adult mental health services (AMHS). The transition from CAMHS to AMHS should be purposeful and planned [1]. After studying a cohort of service users, in six mental health trusts in England, during a 12 month period, Singh et.al, Paul [2] described a successful transition as one that is well planned, with a mutual exchange of information and continuity of treatment. A planned transition is optimized by including family members, and caring for patients’ psychosocial and medical needs in an inclusive and individualized process. However, in the study by Singh et al., fewer than 5% of 154 young people with a mental health disorder experienced a satisfactory transition.

Anorexia nervosa (AN) is an eating disorder characterized by the inability to maintain a minimally normal weight, a devastating fear of weight gain, dietary habits that prevent weight gain, and an overvaluation of body shape and weight [3]. Often, patients have poor insight and do not regard themselves as ill [4, 5]. AN is often a long-lasting illness with severe consequences for the individual and the family. Parents are regularly involved in the treatment of eating disorders in CAMHS in Norway and they often have an active part in meal support at home. Family based therapy for eating disorders as described in the Maudsley model (Treatment Manual for Anorexia Nervosa Second Edition A Family-Based Approach.

James Lock and Daniel Le Grange) has not been implemented in Norway. Continuity of care in the transition from CAHMS to AMHS are especially important for many patients with eating disorders [6–8]. Dimitropoulos, Tran [9] found that patients suffering from AN often have an ambivalent view of their own illness and struggle with motivation for recovery, which makes the transition between services a demanding process.

It is therefore important to facilitate a successful transition from CAMHS to AMHS for patients’ well-being and recovery [2, 10–13]. Poorly planned transition between CAMHS and AMHS has potentially serious consequences for patients with AN (e.g., decreased psychosocial functioning and autonomy as well as a risk of prolonged illness, progression, and chronicity [14]. Clinical guidelines recommend that the timing of the transition should be determined in collaboration with patients and parents, due to the large variation in maturity levels [5]. Many patients with AN have a fear of maturity and may be less ready for AMHS than young people with other mental health problems [15]. Child and adolescent mental health services in Norway have the opportunity to maintain treatment responsibility until the patient is 23 years old, and thus, offer patients a transition that is timed according to their developmental needs, as recommended. Despite this opportunity, services often fail to collaborate and implement optimal transition practices. For example, when patients are 18 years old, CAMHS tend to terminate the treatment plan instead of planning a transition to AMHS [16].

Previous studies have mainly emphasized organizational and structural elements in the transition process from CAHMS to AMHS [11, 12]. However, recently Mulvale et.al [17] conducted a systematic review examining differences in treatment philosophies in CAMHS and AMHS. In this review, three descriptive themes were contrasted: (1) developmental versus diagnostic approaches, (2) focus on social contexts and the importance of family involvement versus an individual approach, and (3) protectiveness versus responsibility.

There is still little knowledge of what a satisfactory transition should comprise for patients, parents, and clinicians [11, 12, 18]. Few studies have explored experiences in the transition from CAHMS to AHMS for mental health service professionals, and to our knowledge, none focused on patients with AN in a mental health care setting or included school nurses. School nurses are important for early detection and intervention of patients with AN [19] To achieve an in-depth understanding of the transition process from CAMHS to AMHS for patients with AN, the professionals’ perspective is important. This perspective provides the opportunity to see the transition from the health care providers` perspective, as well as from the experiences provided by professionals’ relationships with multiple patients with AN and their parents. We included clinicians from different professions with experience of treating young people with AN. General practitioner (GP), school nurses, psychiatrists, psychologists, and psychiatric nurses were represented. We aimed to explore the experiences of professionals concerning the transition from CAMHS to AMHS in a public mental health care setting.

## Methods

In this explorative qualitative study, we used multistep focus group interviews and in-depth interviews to describe eight health care professionals’ experiences with the transition from CAMHS to AMHS for people with severe eating disorders. One former service user and one family member with service use experience were part of every step during the research process. The multi-perspective research group was important in forming the research questions, designing the interview guide, conducting the focus group interviews, and analyzing the transcriptions.

### Data collection

The first author (VL) conducted the semistructured interviews with the last author (JIR). They both have experience as professionals in the general mental health care system in Norway. Two individual interviews and one multistep focus group interview were conducted with the focus on exploring professionals’ experiences with treating people with severe eating disorders in the transition from CAMHS to AMHS. A thematic interview guide was developed by the research group and used as a supporting document (see Additional file 1). Written consent was obtained from all participants. The interviews were audio-recorded and transcribed verbatim by the first author.

Multistep focus groups are viewed as a beneficial method when working with service users in research [19]. The purpose of a multistep focus group interview is to explore the research questions through dialog and reflection. Repeated meetings give the participants the opportunity to inspect and challenge their own and others’ experiences in a dialectic way. The interview process is interactive; the moderators understanding is ever-changing and affects their awareness of the subjects, topics, and nuances in the interviews [20, 21] Accordingly, one focus group met two times. Each interview lasted approximately 1 h and a half. The research group read and discussed the transcripts after each interview, and this created the foundation for the summary distributed to the professionals in the focus group for further deliberation on the transition from CAMHS to AMHS.

After reading through the transcripts, the research group decided to differ from the recommendation to conduct three or four focus group interviews, and instead, conduct individual interviews to facilitate a more in-depth understanding of the experiences.

The first author conducted two qualitative semi-structured interviews, each lasted 1 h and a half. A professional, currently working in CAMHS with experience from AMHS, and a professional with a GP perspective were included. This decision derived from a hermeneutic understanding that values the moderators’ evolved knowledge and experiences from the pre-interviews. This added important aspects to the material, as neither of the other focus group participants had experience of working as a GP or as a psychologist in an out-patient CAMHS setting.

### Data analysis

The research group read the material several times to validate and discuss the results [22]. Data were analyzed using Malterud’s (2012) four steps systematic text condensation. i), the research group read the interviews several times to form an overall impression of the material. ii), then texts that belonged together were grouped and encoded. iii), decontextualized selection of meaning content from the created codes were assembled to reveal aspects of the professionals’ experiences. iv), the material was conceptualized to authentic quoting that represented the validity and wholeness of the original context. To help organize and categorize the transcriptions, we used the computerized program NVivo 11 (QSR International Pty Ltd.).

### Participants

Recruitment for the study took place between February 2018 and April 2018. All participants were recruited from the South-Eastern Norway Regional Health Authority. The participants consisted of eight mental health professionals (seven women and one man) with experience in treating patients with AN and the transition from CAMHS to AMHS. General practitioner (GP), school nurses, psychiatrists, psychologists, and psychiatric nurses were represented to obtain diverse perspectives of the transition process. To ensure that we included experienced professionals, we contacted different units from in-and-outpatient’s treatment facilities seeking complexity and depth of thought. For decades school nurses in Norway have received training to work with adolescents with AN. Using snowball sampling, we recruited clinicians with different experiences of inpatient and outpatient treatment. In addition to having experience with treating eating disorders, three participants had experience coordinating treatment and collaboration between different hospitals and departments.

### Ethical aspects

The study was approved by the data protection authority at Oslo University Hospital (OUS; 2016/19732). The study was performed in accordance with the Declaration of Helsinki and evaluated by the Regional Committee for Medical and Health Research Ethics for the Southeast Region of Norway (2016/1259).

## Results

In the present study, we identified four themes from the interviews with professionals in relation to the barriers experienced in the transition process by patients with AN and by the varied professionals: (1) Different treatment cultures describe how mental health professionals experienced differences in how to involve parents and expectations for patients’ with AN’s autonomy and responsibility; (2) mistrust between CAMHS and AMHS describes the attitudes between CAMHS and AMHS; (3) clinicians` factors describe the level of competence, security, and confidence experienced by the professionals; and (4) lack of trust between services and not enough focus on building a new alliance in AMHS negatively influences the transition.

### Different treatment cultures

The professionals consistently emphasized the difference in treatment cultures between CAMHS and AMHS. The difference is related to the differing involvement of family members and the way patients with AN are expected to take more responsibility for their own health in AMHS than in CAMHS.

#### Involvement of parents

In CAMHS, parents are recognized as an important part of patients’ recovery process. In the focus group, professionals stated, “In CAMHS, the parents are involved from the first meeting.” Some AMHS professionals described fewer resources and less time to involve parents in the treatment process. Moreover, the AMHS professionals found it difficult to prioritize collaboration with parents, and often felt insecure about how to use information provided by parents. A consequence could be that patients’ parents feel less listened to and lose trust in the mental health care system. AMHS professionals acknowledged the benefits of family involvement, and were aware of treatment guidelines, but they found it difficult to implement them in practice. This is often “due to treatment culture and resources,” as one professional in the focus group said. Family members were described as often unprepared for the change of roles in AMHS versus CAMHS. After patients turn 18, they often choose not to involve their parents in treatment. AMHS professionals found this inadvisable and indicated that they want to facilitate interaction between patients and parents. One professional stated, “I try to encourage the patient to include the parents.” AMHS clinicians wanted to involve parents when it is important and appropriate. However, “when you are turning 18 years of age, maybe it is good for you not to be that close with your parents,” as one professional in the focus group said. The professionals understood that being a parent is challenging. However, they described no focus and guidance for how to prepare parents for the transition: “We have experienced that the parents don’t manage and back off,” a professional said.

#### Patients’ responsibility and autonomy

The professionals described that the patient’s age (18 years) normally defines whether patients should be treated in CAHMS or AHMS. According to the professionals, patients are expected to be more responsible for their own health and treatment when they are 18 years old. When transferring patients from CAMHS to AHMS, the professionals emphasized the importance of the patients’ level of maturity and readiness for treatment in AMHS: “The adolescent’s age is around 16–17 years when [they are] finished in CAMHS. You cannot just define that you are old enough and well enough to be an adult, because many are not.” They described it as natural to give patients a higher degree of responsibility for their own health in AMHS. However, the culture difference between how CAMHS and AMHS presume patient self-sufficiency is a hindrance during the transition process. In addition, AMHS traditionally have fewer resources to follow up with patients, as one professional described: “We are very dependent on cooperation with the patients. I think that—the responsibility we put on the patient with AN - feels overwhelming in the transition period.” Although the professionals had examples of good transitions, they consistently described a lack of systematic follow-up and ability to assess individual needs during the transition process. Moreover, the professionals often experienced patients’ ambivalence regarding treatment. Too much responsibility too soon often resulted in relapse. The professionals in the focus group emphasized the importance of being aware of patients’ ambivalence to treatment as a symptom of the AN disorder. CAMHS spend a lot of time trying to motivate patients to continue treatment in AHMS. A therapist stated, “Many have to reflect on how the anorexic patient relates to concepts like autonomy and voluntary treatment—cause many anorexic patients just want to be left alone with their symptoms.”

### Mistrust between CAMHS and AMHS

Although CAMHS and AMHS collaborate and work together during the transition process, CAMHS professionals described transferring patients as difficult. CAMHS professionals are unaware of what kind of care the patients with AN will receive when they enter AMHS. Disregarding the CAMHS long relationship with the patient, professionals in AMHS make their own assessment of the patients when entering AMHS. From the CAMHS` professional’s perspective, it can be difficult to refer a patient you have worked with for a long time to a system you do not trust. “We as a system don’t play on the same team. It is a lack of trust between us.” One CAMHS professional described: “When I asked if I could talk to someone in the evaluation team, they answered that I have to submit a referral. Many do not discuss matters in advance.” CAMHS professionals described a focus on ending the therapeutic relationship rather than transitioning patients to AMHS. This closure was mainly due to a lack of knowledge of what they could expect from the AMHS concerning treatment.

The professionals experienced a lack of mutual understanding of each other’s systems and ideologies of treatment: “They called me from CAMHS, and I could feel they thought—that poor girl who is gone over to the wolves in AMHS.” This makes the establishment of a therapeutic relationship more difficult for AMHS clinician. As they put it: “Maybe it would be easier if CAMHS had prepared them for what is to come instead of give out uncertainty and you poor little one.” From the AMHS perspective, the professional described it as if CAMHS providers often were mothering the patient with AN, and did not give the patients responsibility for their own lives and health, for example concerning eating or follow up treatment.

Some professionals described that CAHMS and AHMS clinicians have different treatment approaches. CAMHS clinicians focus on meeting patients with a holistic approach and flexibility to compensate for the patients’ lack of function and motivation. AMHS clinicians described a stricter diagnostic focus, and they emphasized the patient’s own motivation and responsibility for recovery from their AN*.* In AHMS, patients’ individual freedom seems to be the focus. Consequently, patients become more in charge of their own life and health. A professional stated, “CAMHS want to work from a more contextual approach while AMHS have a more rigid medical understanding.” If the patients are too ill and do not follow up with treatment appointments (or are not motivated enough), they are discharged: “So, if they don’t meet for appointments, they are discharged, and (the patients) experience that they aren’t acknowledged by the professional. So, the system could be a factor in obtaining and amplify the symptoms.”

### Therapist factors

When treating patients with AN, professionals experience a need for competence on many levels. However, despite having this competence, clinicians can find it difficult to trust themselves. This lack of confidence has a negative effect on patients’ transition from CAMHS to AMHS.

#### Competence

Due to the complexity of treating patients with AN, the professionals described a need for a high level of competence. They need to be highly skilled in building relationships and in different aspects of having AN: “These adolescents are so sick and have complicated comorbid conditions.” The professionals described how they felt their personal attitudes influenced patients’ motivation for treatment and hope for recovery. They emphasized that they had to be present, understanding and use sensible formulations not to be perceived as dismissive. Additionally, they had to communicate their competence and clinical skills to the patients.

Furthermore, the professionals described patients with AN as sensitive and in need of a close relationship with a competent therapist. Patients are often left with a sense of being too difficult: “Adolescents are concerned with what and how we can help them. We have to be ‘in the same boat’ and show them that we care.”

#### Clinicians` feelings of security

The professionals described it as difficult to believe in their own competence in meeting the needs of patients with AN. The professionals often felt a need for support and supervision to have confidence in their own decisions, to provide patients with the level of security they need. The professionals had experienced colleagues who claimed feeling incompetent during the transition period (“I have no knowledge about this”) and found that easy to understand. The professionals were also influenced by patients’ behavior and attitudes. AMHS professionals experienced that patients and parents have high expectations for treatment, much higher than the professionals have the capacity to fulfill. This influences the way patients are met during the transition period: “My experience is that these patients have so pronounced expectations combined with their own uncertainty that it’s easy to get insecure as a therapist.” A professional said, “I have more experience helping AMHS clinicians with feeling confident and able to manage the pressure from the patients. Just by saying that they do a good job, they calm down.”

Professionals, based on their experiences, think that the patients need proof that they really care for them. Some patients even feel a need to test the professionals to see if they really care. This was described as challenging by the professionals: “This is a group of patients that is challenging to work with. They have a grip on you, and, sometimes, I go home and think to myself, when am I going to be finished with this one?” They described some degree of anxiety about when patients undergo a bad period and/or reject the treatment offered. A consequence is that the patients can be discharged too soon and maintain their destructive way of living: “A patient once said to me, ‘Do you know why I got ill again? Cause they stopped treating me, and I wanted to get back to my therapist.” The professionals emphasized that in mental health care units, nurses and doctors have basic knowledge about treatment of AN due to their profession. Still, it can be difficult for them to rely on their own expertise, and they often want to transfer patients to specialized eating disorder treatment units.

### Transfer of alliance

The professionals experienced that there is a lack of transfer of alliance and trust between systems, clinicians, and the patients. The professionals assessed the change in clinicians as an important element in the transition, because patients are especially vulnerable to changes in close relationships. This factor complicates the transfer for an unsure, ambivalent patient with AN: “We don’t manage the transfer of therapeutic alliance and level of trust the adolescent needs.” To try to compensate for this lack of a safe meeting point in the transition to AMHS, the school nurse or someone the patient relies on in CAMHS makes a connection with AMHS: “We often make a phone call to inform AMHS what the essential problem is, because patients view AMHS’ approach as scary.” The professionals experienced that patients have to elaborate their story several times during the transition, which has a negative impact on the patients’ motivation for treatment. CAMHS and school nurses try to bridge the information flow, but more often than not, the patients have to elaborate anyway.

The professionals described that it seems difficult for the patients to start therapy in AMHS. It appears difficult to achieve a good therapeutic relationship, as the patients easily feel rejected. The consequence is often that patients drop out of treatment, “and they end up back with us and the GP,” as a school nurse said.

The professionals experienced that the patients have a pressing need to be acknowledged, and it is important for them to feel perceived for more than their eating disorder. When the patients are given a treatment plan that does not meet their expectations, they feel rejected: “They are often met with ‘now we have ten sessions`—and when they know that, this approach don’t meet their needs for more long-term therapy.” The experience is that many patients drop out of treatment and relapse.

## Discussion

The transition period between CAMHS and AMHS has been identified as an especially critical time for patients with AN. Poorly planned transition between CAMHS and AMHS has potentially serious consequences for patients with AN (e.g., decreased psychosocial functioning and autonomy as well as a risk of prolonged illness, progression, and chronicity).

The present study revealed barriers, classified into four categories, concerning the transition process between CAMHS and AMHS for patients with AN. (1) Different treatment cultures that describe differences in how parents are included in CAMHS and AMHS and how patients feel unprepared for the increased level of individual responsibility in AMHS; (2) mistrust between CAMHS and AMHS that can create a lack of collaboration and predictability for the patients’ transition; (3) individual clinicians factors, e.g., lack of professional confidence, that can influence continuity of care for patients; and (4) lack of trust between services and not enough focus on building a new alliance in AMHS negatively influence the transition.

CAMHS and AMHS have different approaches for involving parents in patients’ treatment. This creates diversity in how parents can support patients’ recovery process and complicates the transition process. Pereira et.al [23] explored the role of therapeutic alliance in family therapy for adolescent patients with AN, and found that a strong alliance with parents prevents dropout. Parents who are included in the patient’s recovery process have a positive impact on the patient’s compliance and contribute positively during the transition process. Dimitropoulos et.al [9] found that professionals perceive significant barriers during the transition if the parents are not involved in the process, as the patients often referred to the fact that they felt dependent on parents` external support to continue treatment. In many situations, in the clinic or at home, a patient with AN will not eat unless supported by an adult. Consequently, it is important for the patient’s recovery that the treatment system understand the parents’ significant role in the treatment.

In this study, according to the professionals, parents seemed unprepared for the changes of roles in AMHS and the parents` reduced influence on the treatment offered. The professionals perceived parents as needing more information that is viewed as important for the patient’s recovery process. Dixon et.al [24] found that parents feel left out of the transition process, and that this causes them distress. Furthermore, Dimitropoulos et.al [9] found that professionals experienced the transition process as shocking for the parents, and that they seem unprepared for their role in the shift. Although the present study showed that professionals experienced that patients have different needs when it comes to their parents` involvement in the transition process, we recommend that parents should be involved in a systematic way. This might facilitate the support patients need to manage a successful transition process.

In this study, the professionals experienced the age boundary (18 years) as the decisive element for the transition from CAMHS to AMHS. When planning the transition from CHAMS to AMHS, patients’ maturity is often not sufficiently considered. Patients with AN often fear “maturity” and are less ready for transitioning to AMHS [15]. The sudden change in treatment philosophy from a “protective” approach to a “responsibility” approach may be stressful for patients and parents. Professionals assessed patients with AN as lagging behind their peers in the ability to make decisions benefitting their recovery as required in AMHS [9] This makes it more difficult and challenging for patients with AN to stay motivated [25]. This present study shows that clinicians recognise that patients and parents may struggle with the different treatment approaches in CAHMS and AMHS and that this can be a hindrance for an optimal transition process. Patients with AN would probably benefit from a longer and more carefully planned transition period that takes into consideration the patients’ need for a gradual adoption of adult responsibilities and the patients need to be in charge of their own health and life. This study revealed that professionals experienced a lack of trust and understanding between CAMHS and AMHS that might deteriorate the transition process. A consequence is that CAMHS often focus on ending the relationship rather than transferring patients to AMHS. This could be because CAMHS and AMHS have different treatment philosophies and are organized differently [9, 26, 27]. CAMHS treatment philosophies are based on a developmental approach that involves families. AMHS are based on a clinical and diagnostic focus that emphasizes individuals’ own responsibility for their health and autonomy ([17]. McLaren et.al [28] emphasized cultural differences that embody mutual misperceptions and attitudes toward each other. Furthermore, Appleton et.al [29] identified that young patients were not referred to AMHS because CAMHS professionals did not believe that the patients would meet the inclusion criteria or thought that AMHS did not have the necessary expertise. In the present study, mistrust between the two services implied difficulties for patients with AN and parents for trusting and engaging in the new treatment service. This may negatively influence the already difficult transition.

A high level of professional competence is crucial for managing a successful transition process for patients. This is challenging for professionals, as AN is a complex disorder, and patients have diverse needs during this period. The professionals described how clinicians’ sense that patients expect them to be experts on AN. This is in line with a study by Gulliksen et.al [30], who found that patients appreciated receiving treatment from professionals who are specialists in eating disorders (EDs), and that the clinicians expressed self-confidence. This in contrast to the findings in a study by Pettersen and Rosenvinge [31], who found that patients rated specific knowledge about eating disorders as less important than having a safe and supportive relationship with the therapist.

Due to the ambivalence many people with AN have toward treatment, it is often challenging for professionals to stay in an open and reflective dialog. This can increase the possibility that professionals overplay their authoritarian position and push patients adversely. Loos et.al [32] reported that patients often value “parental-like” support in the therapeutic relationship. In the present study, the professionals often experienced that patients wanted a more personal and caring therapist role than the professionals were able to provide during the transition period. To establish a good alliance with patients and parents, professionals might need external support and supervision during the transition period.

The present results indicate that there is an absence of an alliance and trust between the systems, clinicians, and the patients. A lack of insight and understanding between the services can explain why CAMHS often miss the opportunity to prepare patients for the transition from CAMHS to AMHS. This is in line with Singh et.al [2], who found that sensitivity and consideration for patients are not always prevalent during the transition process. A good relationship with the treating clinician is significant for recovery in CAMHS and AMHS. However, the importance of a solid relationship becomes even more salient when patients enter AMHS [9, 33]. Patients go through significant changes in this period and are dependent on a close relationship with their clinicians to continue treatment. This study showed how complex the transition process is for both patients, parents and professionals. A comprehensive focus of the on the matters identified in this study would probably increase the chance of patients staying in treatment during the transition period.

### Limitations

The professionals included in the study treated patients with AN in a capital area in Norway, with easier access to specialist ED services. Professionals in rural areas might face other challenges than the study participants elaborated. Our findings are not generalizable for all transitions from CAMHS to AMHS. Instead, they describe a diverse, but small sample of experienced clinicians’ perspectives, which provide an overview of important aspects of transition.

## Conclusion

Professionals involved in the care of young people with AN, experience barriers in the transition from CAMHS to AMHS. These relate to professionals’ own role, and how CAMHS and AMHS collaborate regarding the patients’ recovery process. These barriers complicate the transition for the patients and their parents.

Patients and parents are not prepared for the differences between CAMHS and AMHS. Professionals should be aware of how unprepared they are, and discuss with them possible difficulties in the transition process. We suggest developing written procedures to facilitate a more satisfactory transition process for patients with AN.

## Supplementary information

**Additional file 1.** Interview guide professionals.

## Data Availability

The datasets used and/or analyzed during the current study are available from the corresponding author upon reasonable request.

## Abbreviations

CAMHS: Child and adolescent mental health services
AMHS: Adult mental health services
AN: Anorexia nervosa
