# Supplementary material for: Mental health professionals’ experiences transitioning patients with anorexia nervosa from child/adolescent to adult mental health services: a qualitative study
Source: BMC Health Serv Res. 2020 Sep 21;20:891. doi: 10.1186/s12913-020-05740-2 (PMC7507603; doi:10.1186/s12913-020-05740-2)
Supplement: Supplementary file 1 — Additional file 1. Interview guide professionals. [file 12913_2020_5740_MOESM1_ESM.docx]

Interview guide professionals

# Theme: patients and parents/carers

Based on your experience, how do you assume the patients experience the transition period?

- Examples?

How do you expect the parents experience the transition period?

- Examples?

In your opinion, what are the important aspects of a good transition? Which factors are important for patients and parents?

Based on your experience, how do you evaluate if the patients are ready for the transition to AMHS?

- How do you evaluate the patient’s maturity and readiness for transition? (With emphasis on age)
- Examples?

# Theme: actions

Based on your experience, what are the most important aspects of a successful transition from CAMHS to AMHS?

Working with transitions from CAHMS to AMHS, can you describe your routines regarding planning and follow- up?

Based on your experience, how would you describe the patient’s role in the transition?

# Theme: your self

How do you think your relationship with the patient affects the transition process?

- What can influence the transition in a positive/ negative way?
- When thinking about transitions, have you yourself experienced any reactions in the transition process? (feelings)

# Theme: the others

Based on your experience, how would you describe the collaboration between CAMHS/AMHS?

Which specific elements would you emphasize?

How would you describe the differences in treatment approaches between CAMHS/AMHS?

- How are these verbalized to the patients/parents?
